# Recent advances in mitochondrial fission and fusion in diabetic cardiomyopathy

**DOI:** 10.3389/fcvm.2026.1897303

**Published:** 2026-09-04

**Authors:** Jinjin Hao, Xiaochen Zhang, Xueying Tian, Jingying Su

**Affiliations:** Department of Geriatrics, Taicang Loujiang New City Hospital (Ruijin Hospital, Taicang), Taicang, China

**Keywords:** diabetic cardiomyopathy, mitochondrial dynamics, mitochondrial fission, mitochondrial fusion, mitophagy

## Abstract

Diabetic cardiomyopathy (dCM) is characterized by myocardial dysfunction in diabetes and reflects interacting metabolic, redox, calcium, inflammatory, fibrotic, and microvascular disturbances. Mitochondrial fission and fusion are not merely morphological endpoints; when uncoupled from mitophagic clearance and cristae maintenance, they contribute to energetic failure and cell-specific cardiac injury. This review synthesizes evidence from human diabetic myocardium and dCM-specific experimental models, while distinguishing direct fission-fusion evidence from broader mitochondrial quality-control findings. We summarize how glucolipotoxicity, impaired energy sensing, calcium entry, mechanosensing, innate immunity, epitranscriptomic regulation, and ubiquitin editing converge on dynamin-related protein 1 (DRP1)/ fission 1 (FIS1)/ mitochondrial fission factor (MFF) and mitofusin 1 (MFN1)/ mitofusin 2 (MFN2)/ optic atrophy 1 (OPA1) pathways. We further compare consequences in cardiomyocytes, cardiac fibroblasts, and coronary microvascular endothelial cells, including ATP depletion, oxidative stress, regulated cell death, fibrosis, and perfusion injury. Candidate interventions are organized according to whether they restrain pathological fission, restore MFN/OPA1-dependent fusion, or normalize mitophagic flux. Despite strong cellular and rodent evidence, direct clinical validation and pharmacodynamic biomarkers remain limited. Future progress will require human myocardial phenotyping, single-cell and spatial analyses, *in vivo* measurement of mitochondrial dynamics, cell-selective delivery, and standardized flux-based endpoints. The therapeutic goal should be restoration of adaptive mitochondrial dynamics rather than indiscriminate inhibition of fission or promotion of fusion.

## Graphical Abstract

**Figure:**
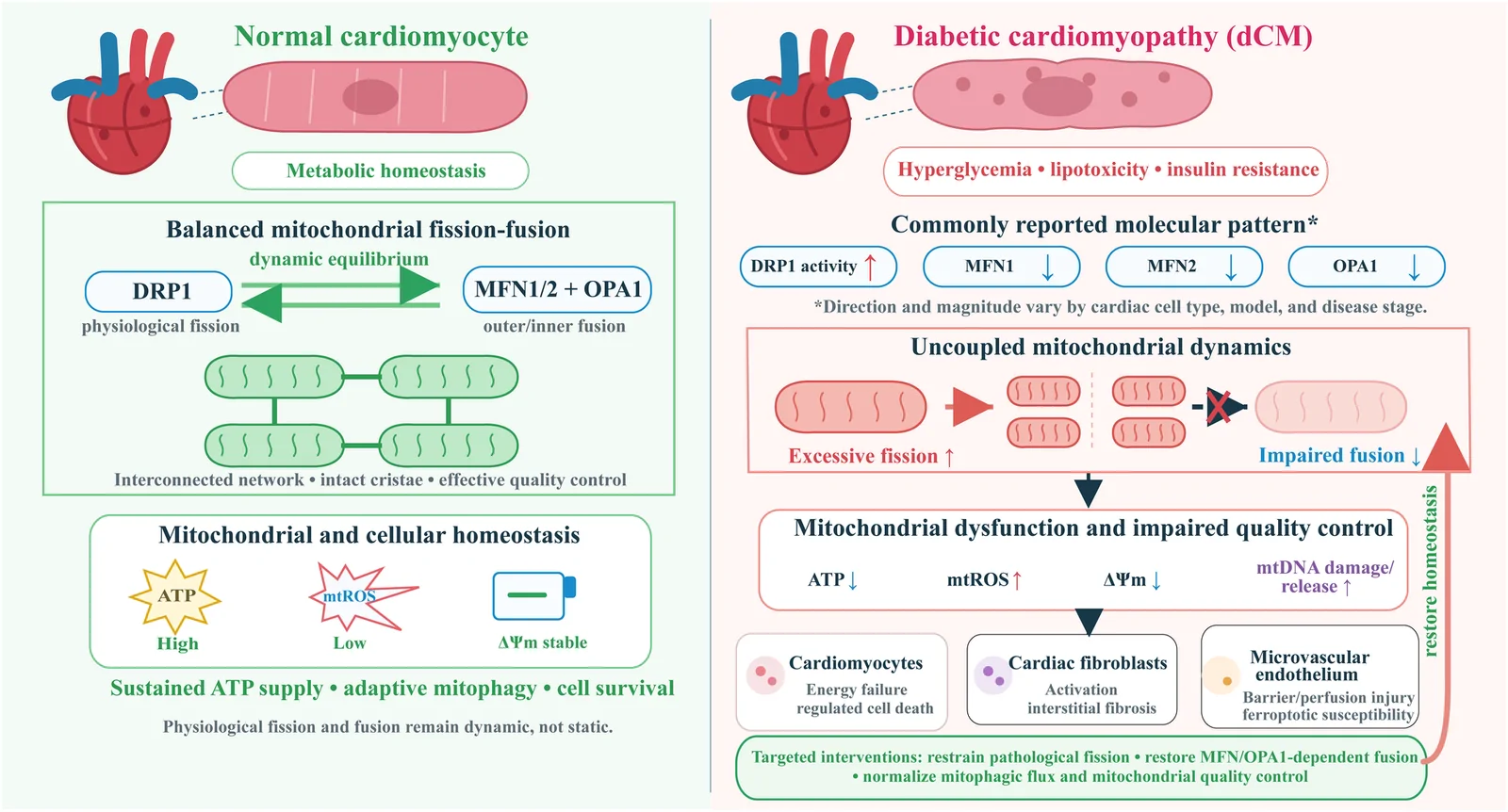

Context-dependent remodeling of mitochondrial fission, fusion, and quality control in diabetic cardiomyopathy. In the normal cardiomyocyte, physiological DRP1-dependent fission is coupled to MFN1/MFN2- and OPA1-dependent fusion and mitophagic clearance, thereby maintaining an interconnected mitochondrial network, ATP production, redox balance, and mitochondrial membrane potential. In dCM, hyperglycemia, lipotoxicity, insulin resistance, calcium dysregulation, and inflammatory stress commonly increase DRP1/FIS1/MFF-associated fission and impair MFN/OPA1-dependent fusion, although the direction and magnitude of these changes vary by experimental model, cardiac cell type, and disease stage. Uncoupling of fission, fusion, and mitochondrial quality control promotes mitochondrial fragmentation, reduced ATP production, increased mitochondrial ROS, loss of membrane potential, and mtDNA damage or release. These abnormalities contribute to cardiomyocyte energetic failure and regulated cell death, cardiac fibroblast activation and interstitial fibrosis, and coronary microvascular endothelial injury. Interventions that selectively restrain pathological fission, restore MFN/OPA1-dependent fusion, and normalize mitophagic flux may re-establish mitochondrial homeostasis and improve cardiac function (6, 12, 14–17, 20–24, 27, 28, 31, 42, 46, 47). dCM, diabetic cardiomyopathy; DRP1, dynamin-related protein 1; FIS1, fission 1; MFF, mitochondrial fission factor; MFN1/2, mitofusin 1/2; OPA1, optic atrophy 1; mtROS, mitochondrial reactive oxygen species; ΔΨm, mitochondrial membrane potential; mtDNA, mitochondrial DNA.

## 1 Introduction

Diabetic cardiomyopathy (dCM), increasingly discussed within the broader concept of diabetic myocardial disorder, refers to structural and/or functional myocardial abnormalities occurring in people with diabetes. Although it was historically defined after excluding coronary artery disease, hypertension, valvular disease, and obesity, contemporary frameworks recognize that diabetes commonly interacts with these comorbidities rather than acting as an isolated cause (1). The phenotype frequently begins with metabolic inflexibility, impaired relaxation, and subclinical microvascular dysfunction, and can progress to fibrosis, hypertrophy, and heart failure. These changes arise from interacting disturbances in substrate use, insulin signaling, calcium handling, redox balance, inflammation, and extracellular-matrix remodeling rather than from a single linear pathway (2–5).

Mitochondrial abnormalities are consistently observed in diabetic hearts, but the relevant defect is not adequately captured by the generic label ‘mitochondrial dysfunction.’ Myocardial samples from patients with type 2 diabetes demonstrate impaired respiration, abnormal mitochondrial architecture, and reduced fusion-related proteins (6). Human multi-omics data further link lipid metabolic remodeling, defective mitophagy, and extracellular-matrix activation (7), whereas single-cell RNA sequencing in high-fat diet (HFD)/ streptozotocin (STZ)-induced diabetic mice identifies cell-type-specific profibrotic communication networks (8). Mitochondrial dynamics encompasses two coordinated processes: fission, in which one organelle divides, and fusion, in which mitochondrial membranes and contents merge.

This narrative review therefore has three specific objectives. First, it distinguishes evidence obtained in dCM or diabetic human myocardium from mechanisms extrapolated from ischemia-reperfusion injury, doxorubicin cardiotoxicity, or non-cardiac tissues. Second, it maps dCM-specific upstream signals to the core fission-fusion machinery and to cell-type-specific downstream phenotypes. Third, it grades therapeutic candidates by experimental maturity and identifies the measurements needed for translation. We argue that therapeutic strategies should target the overall balance of mitochondrial turnover instead of one-sided inhibition of fission or fusion, as the functional demand of mitochondrial dynamics varies across cardiac cell types and disease stages.

## 2 Mitochondrial fission and fusion in dCM

### 2.1 DRP1-dependent mitochondrial fission

Mitochondria undergo continuous mitochondrial fission and fusion cycles to maintain homeostasis, facilitate turnover, and adapt to environmental changes. Fission divides a mitochondrion into smaller, functionally independent units, modulating mitochondrial quantity, morphology, and distribution. Mitochondrial fission is executed principally by the cytosolic GTPase dynamin-related protein 1 (DRP1) after its recruitment to the outer mitochondrial membrane by receptors and adaptors including mitochondrial fission factor (MFF), fission 1 (FIS1), mitochondrial dynamics protein of 49kDa (MID49) and mitochondrial dynamics protein of 51kDa (MID51). DRP1 oligomerization constricts the organelle at sites coordinated with the endoplasmic reticulum and cytoskeleton. Phosphorylation and other post-translational modifications determine its localization and activity; phosphorylation at serine (Ser) 616 is commonly associated with pro-fission signaling, whereas Ser637 phosphorylation often restrains mitochondrial recruitment, although these relationships are stimulus- and kinase-dependent (9, 10).

Physiological fission is indispensable for mitochondrial distribution, segregation of damaged segments, and completion of mitophagy. The pathological feature in dCM is therefore not fission *per se*, but sustained or spatially inappropriate fission that exceeds the capacity for fusion and clearance. Across diabetic cardiomyocytes and hearts, this state is frequently accompanied by increased DRP1 recruitment or Ser616 phosphorylation and increased FIS1/MFF signaling. Importantly, a fragmented image at a single time point cannot by itself distinguish accelerated fission from failed fusion or blocked mitophagic turnover; mechanistic studies should combine morphology with dynamic flux and rescue experiments.

It is worth emphasizing that physiological DRP1-mediated fission is indispensable for mitochondrial transport and damaged fragment clearance. Because basal DRP1-dependent fission supports mitochondrial distribution and damaged-segment segregation, therapeutic strategies should selectively restrain pathological DRP1 activation rather than assume that complete and sustained DRP1 inhibition is uniformly beneficial.

### 2.2 MFN1/MFN2- and OPA1-dependent mitochondrial fusion

Mitochondrial fusion integrates two or more mitochondria into a larger, functional organelle, promoting mitochondrial integrity, energy production, and metabolic flexibility. Outer-membrane fusion is mediated by mitofusin 1 (MFN1) and mitofusin 2 (MFN2), whereas OPA1 coordinates inner-membrane fusion and cristae architecture. Fusion allows exchange of matrix metabolites, proteins, and mitochondrial DNA, thereby buffering focal injury and supporting oxidative phosphorylation. MFN2 also participates in endoplasmic-reticulum-mitochondria contacts and calcium transfer, so changes in MFN2 can alter both network morphology and organelle communication (9, 10).

Human diabetic myocardium shows reduced MFN1 expression together with mitochondrial fragmentation and respiratory impairment, whereas experimental dCM models additionally implicate loss of MFN2- and OPA1-dependent fusion in cristae disorganization and contractile dysfunction (6, 11, 12). Insulin can acutely stimulate OPA1-dependent fusion through protein kinase B (Akt)- mammalian target of rapamycin (mTOR)- nuclear factor kappa-light-chain-enhancer of activated B cells (NF-κB) signaling in cardiomyocytes (11), whereas chronic insulin resistance removes this adaptive response. Cardiomyocyte-specific manipulation of MFN2 in diabetic mice further supports a causal role for impaired fusion, but the phenotype should be interpreted together with MFN2-dependent calcium and mitophagy functions (12). Functionally, this machinery links morphology to the injury pathways discussed below. DRP1/FIS1-driven fragmentation can increase electron-transport stress, ROS leakage, and outer-membrane permeabilization, whereas OPA1 loss destabilizes cristae and facilitates cytochrome c release. Because fission also segregates damaged segments for mitophagy, either excessive fission or failure to couple fission to lysosomal clearance can deplete respiratory reserve and promote regulated cell death (9, 10).

### 2.3 Context dependence of mitochondrial remodeling: model, cell type, and disease stage

The shift of mitochondrial fission-fusion remodeling varies with diabetes duration, subtype, metabolic burden and cardiac cell types. Common diabetic animal models carry inherent bias: streptozotocin models mimic insulin deficiency and hyperglycemia, db/db or ob/ob mice recapitulate leptin-related obesity and insulin resistance, while high-fat diet combined with low-dose streptozotocin reproduces glucolipotoxicity and lipotoxicity. No single animal model fully recapitulates human diabetic cardiomyopathy; therefore, study models and metabolic characterization should be clearly stated (5).

Evidence from human myocardial samples remains limited yet more translational. Three-dimensional electron microscopy has confirmed spatially heterogeneous mitochondrial ultrastructural lesions in dCM, and human cardiac multi-omics further linked lipid remodeling, defective mitophagy and extracellular matrix activation (7, 13). These findings refute the oversimplified universal rule of “enhanced fission/suppressed fusion”. We propose a refined model: chronic diabetic stress narrows the adaptive window of mitochondrial dynamics, decoupling fission from mitophagic clearance, impairing fusion and cristae repair, and disabling metabolic matching between substrate supply and cardiac contractile demand.

## 3 dCM-specific regulators of mitochondrial dynamics

### 3.1 Glucolipotoxicity and AMPK/SIRT1-dependent energy sensing

The diabetic heart is exposed to simultaneous glucose excess, elevated fatty-acid delivery, insulin resistance, and incomplete lipid oxidation. This substrate pressure increases acyl intermediates and mitochondrial reducing equivalents, weakens AMP-activated protein kinase (AMPK) signaling, and sensitizes the fission machinery. In myocardial microvascular endothelial cells, empagliflozin activates AMPK and restrains DRP1-dependent fission, linking an established cardiometabolic drug to mitochondrial network protection in a dCM context (14). In cardiomyocytes, restoration of SIRT1-PGC-1α signaling by melatonin likewise limits DRP1-dependent fragmentation and improves bioenergetic and contractile indices (15).

Recent work has expanded this pathway beyond canonical kinases. Regulator of calcineurin 1 (RCAN1) promotes lipid accumulation and mitochondrial fission in diabetic hearts, whereas RCAN1 suppression improves lipid handling and cardiac function (16). Dual specificity phosphatase 26 (DUSP26) is reduced in dCM; restoring it interrupts FAK-ERK signaling, decreases pathological fission, and preserves mitochondrial function (17). Two deubiquitinases provide complementary mechanisms for AMPK suppression. Cardiomyocyte ovarian tumor protease 1 (OTUD1) removes K63-linked ubiquitin chains from AMPKα2 at Lys60/Lys379, impeding its interaction with CAMKK2 and thereby reducing AMPK Thr172 phosphorylation. VCP-interacting protein 1 (VCPIP1) deubiquitinates AMPK*γ*1 at lysine (Lys) 234 and disrupts AMPK α-*γ* subunit assembly (18, 19). These findings place ubiquitin editing of the AMPK complex upstream of mitochondrial remodeling and identify a mechanistically coherent target class for dCM.

### 3.2 Calcium overload and mechanosensitive DRP1 activation

Diabetic cardiomyocytes experience abnormal calcium entry and altered endoplasmic-reticulum-mitochondria coupling. High glucose activates calcium release-activated calcium channel protein 1 (Orai1)-dependent calcium influx, which increases DRP1 activity and contributes to cardiomyocyte hypertrophy and mitochondrial fragmentation (20). Piezo-type mechanosensitive ion channel component 1 (Piezo1) constitutes a mechanosensitive pathway governing the aforementioned molecular machinery. In diabetic mice, cardiomyocyte-specific Piezo1 knockout suppresses ERK-DRP1 cascades, sustains normal mitochondrial morphology, and restores cardiac function (21). These studies are dCM-specific examples of how electrophysiological, mechanical, and metabolic dysfunction converge at DRP1 rather than independent pathways.

The consequences extend beyond morphology. Excess mitochondrial calcium promotes dehydrogenase overload and opening of the mitochondrial permeability transition pore (mPTP); sustained mPTP opening dissipates membrane potential, uncouples oxidative phosphorylation, promotes matrix swelling, increases ROS production, and can precipitate cell death (9, 10). Conversely, excessive disruption of organelle contacts can impair physiological calcium transfer required for ATP production. Thus, interventions should seek to restore the amplitude and timing of calcium exchange rather than abolish endoplasmic-reticulum-mitochondria communication.

### 3.3 Redox stress, innate immunity, and inflammatory cell-death signaling

Mitochondrial fragmentation and redox stress reinforce one another in dCM. Fragmented mitochondria with impaired cristae architecture exhibit less efficient electron transport and greater ROS leakage; ROS then activates stress kinases and further promotes DRP1 recruitment. When membrane integrity is lost, mitochondrial DNA enters the cytosol and activates cyclic GMP-AMP synthase (cGAS)- stimulator of interferon genes (STING) signaling. In obesity-related diabetes, lipotoxicity-induced mitochondrial DNA release drives inflammatory myocardial injury through this pathway (22). It has been shown that leptin treatment maintains OPA1-dependent mitochondrial fusion and inhibits cGAS-STING pathway activation in experimental diabetic cardiomyopathy. This dual effect stabilizes mitochondrial cristae and dampens innate immunity, indicating a mechanistic link between mitochondrial fusion and immune modulation (23).

FIS1 represents another interface between mitochondrial morphology and inflammatory signaling. Lipotoxicity induces FIS1-associated mitochondrial fragmentation and NOD-like receptor family pyrin domain containing 3 (NLRP3)-dependent pyroptosis, whereas FIS1 inhibition reduces inflammasome activation and cardiac injury (24). Importantly, the effects of NF-κB signaling in dCM appear to be context dependent rather than uniformly detrimental. In the GULP1 study, relief of IκB kinase inhibitory protein (IKIP)-mediated inhibition of IκB kinase β (IKKβ) generated a defined NF-κB-dependent increase in OPA1 expression and improved mitochondrial morphofunction (25). In contrast, sustained ubiquitin-specific protease 24 (USP24)-associated NF-κB activation promoted ferroptotic injury (26), whereas RTA 408 reduced pro-inflammatory NF-κB signaling while activating nuclear factor erythroid 2–related factor 2 (Nrf2)-dependent antioxidant responses (27). These apparently divergent effects may reflect differences in upstream regulators, activation kinetics, cellular context, and downstream transcriptional targets. Accordingly, the available evidence supports pathway- and context-selective modulation rather than global activation or inhibition of NF-κB. Collectively, these studies indicate that mitochondrial dynamics can function as both an upstream source and a downstream target of inflammatory signaling.

### 3.4 Epitranscriptomic and ubiquitin-dependent regulation

dCM alters the stability, localization, and assembly of dynamics proteins through regulatory layers that are not apparent from total protein abundance. In cardiac fibroblasts, loss of alkB homolog 5 (ALKBH5) increases N6-methyladenosine (m6A)-dependent YTH N6-methyladenosine RNA binding protein 2 (YTHDF2) phase separation and accelerates notch receptor 1 (NOTCH1) mRNA decay. Reduced NOTCH1 promotes mitochondrial fission, fibroblast proliferation, and cardiac fibrosis; the pathway was supported by diabetic human-heart and experimental data (28). This mechanism is especially important because it demonstrates that fission remodeling in dCM is not confined to cardiomyocytes.

At the organelle surface, phosphoglycerate mutase 5 (PGAM5) perturbs prohibitin 2 (PHB2)-dependent mitochondrial dynamics and worsens hyperglycemia-induced myocardial dysfunction (29). Forkhead box O1 (FOXO1)-mediated suppression of signal transducer and activator of transcription 3 (STAT3) impairs mitochondrial quality control (30), while deubiquitinases such as OTUD1, VCPIP1, and ubiquitin-specific protease 33 (USP33) regulate AMPK activation or autophagy-dependent FIS1 turnover (18, 19, 31). These observations make a strong case for measuring phosphorylation, ubiquitination, proteolytic processing, and complex formation in addition to transcript and total-protein levels.

### 3.5 Cell-specific remodeling in cardiomyocytes, fibroblasts, and endothelial cells

Cardiomyocytes have long been the primary research focus in this field owing to their high mitochondrial density and direct involvement in cardiac contractile dysfunction. In these cells, DRP1/MFN/OPA1 remodeling primarily affects ATP production, calcium buffering, cristae organization, and cell survival. However, a cardiomyocyte-only model cannot explain the microvascular and fibrotic components of dCM.

Cardiac fibroblasts use mitochondrial remodeling to support activation and proliferation; the ALKBH5-YTHDF2-NOTCH1 pathway provides direct evidence that fission can drive diabetic fibrosis (28). In coronary microvascular endothelial cells, AMPK-DRP1 signaling and the USP33-ATG7-FIS1 axis regulate mitochondrial fragmentation, autophagy, endothelial survival, and perfusion (14, 31). Single-cell analysis further suggests that cardiomyocyte subtypes, activated fibroblast states, endothelial subsets, and immune cells participate in distinct ligand-receptor networks during dCM progression (8). Future experiments should therefore use lineage-specific perturbation and cell-resolved readouts rather than inferring global cardiac alterations reflect uniform changes across all cardiac cell populations. Most existing studies prioritize cardiomyocytes, while the mitochondrial regulatory mechanisms of cardiac fibroblasts and microvascular endothelial cells remain understudied. Further cell-specific knockout models are needed to clarify intercellular crosstalk.

## 4 Consequences of fission-fusion imbalance in dCM

### 4.1 Energy failure and oxidative stress

The diabetic heart initially compensates for reduced glucose oxidation by increasing fatty-acid use, but this adaptation raises oxygen demand and can overload the electron-transport chain. Excessive mitochondrial fission isolates individual respiratory units, whereas insufficient fusion blocks metabolite exchange and cristae restoration. Together, these defects reduce respiratory reserve and ATP production. Meanwhile, fragmented mitochondria trigger persistent ROS leakage and collapse of membrane potential. Human myocardial studies and three-dimensional ultrastructural analyses support the coexistence of morphological disorganization and respiratory impairment (6, 13).

ROS should not be considered a downstream marker alone. Oxidative modification of fusion and fission machinery, stress-kinase activation, and mitochondrial DNA damage further destabilize the network. This positive-feedback loop explains why antioxidant interventions may be ineffective unless they also restore substrate handling, organelle quality control, or dynamics signaling.

### 4.2 Apoptosis, pyroptosis, and ferroptosis

Loss of OPA1-dependent cristae integrity facilitates cytochrome c release and apoptotic signaling, whereas persistent DRP1/FIS1 activity promotes outer mitochondrial membrane permeabilization and mitochondrial fragmentation. In dCM, these events occur in parallel with inflammatory and lipid peroxidation pathways rather than isolated cell death cascades. FIS1-dependent fragmentation amplifies NLRP3-mediated pyroptosis (24), while USP24-NF-κB signaling and disturbed mitochondrial lipid metabolism promote ferroptosis (26).

Mitochondrial quality control interventions can block these cascades at multiple regulatory nodes. The microtubule affinity-regulating kinase 4 (MARK4)- acyl-CoA synthetase long-chain family member 4 (ACSL4) axis links diabetes-associated lipid remodeling to increased ferroptotic susceptibility (32). Nicorandil protects the diabetic cardiac microvasculature via a mitochondria-localized AMPK-Parkin-ACSL4 pathway, and cardiomyocyte-specific leucine-rich repeat-containing G-protein coupled receptor 6 (LGR6) restrains ferroptosis by enhancing mitochondrial biogenesis (33, 34). These findings expand therapeutic evaluation endpoints beyond apoptosis and emphasize that mitochondrial dynamics, mitophagy, and phospholipid oxidation should be assessed in combination. Mechanistic studies outside diabetes further show that perturbing DRP1/MFN1/2/OPA1 homeostasis can activate an AMPK-NRF2-FSP1 defense program and reduce ferroptotic susceptibility, whereas MFN2-dependent preservation of fusion limits ferroptosis in myocardial ischemia/reperfusion injury (35, 36). These findings support a functional connection between the dynamics apparatus and ferroptosis but require direct validation in dCM.

### 4.3 Mitophagy dysfunction across models and disease stages

Fission can isolate damaged mitochondrial segments for Parkin-dependent or receptor-mediated mitophagy, whereas fusion can temporarily dilute local damage. Either process becomes pathological when uncoupled from lysosomal flux. The status of mitophagy varies with disease progression. Mitophagic flux in dCM appears to be model-, cell-, and stage-dependent. Both insufficient clearance of damaged mitochondria and excessive depletion of the functional mitochondrial pool have been reported; however, a universal early-to-late sequence has not yet been established. Therefore, measurements limited to microtubule-associated protein light chain 3 (LC3), PTEN-induced kinase 1 (PINK1), Parkin, or autophagosome number cannot determine whether mitophagy is enhanced or impaired. When clearance is insufficient, depolarized mitochondria with impaired respiratory-chain function accumulate, reducing ATP reserve while increasing electron leakage and ROS; conversely, excessive clearance can deplete the functional mitochondrial pool (37–39).

SFRP2 illustrates the need for flux-based interpretation. It improves mitochondrial dynamics, biogenesis, and redox balance, and suppresses apoptosis in experimental dCM, and subsequent work linked secreted frizzled-related protein 2 (SFRP2)- frizzled class receptor 5 (FZD5)-calcineurin signaling to transcription factor EB (TFEB)-dependent mitophagy (37, 38). Mitochondrial aldehyde dehydrogenase 2 also preserves mitochondrial integrity and Parkin-mediated mitophagy via glycogen synthase kinase 3β (GSK3β)-related signaling (39). Experimental designs should combine mitophagy reporters, lysosomal blockade, respiratory measurements, and morphology to determine whether a treatment restores quality-control throughput.

### 4.4 Fibrosis and coronary microvascular dysfunction

Mitochondrial dynamics contributes to dCM remodeling at both the parenchymal and vascular levels. Fibroblast fission supports proliferation and matrix synthesis through the ALKBH5-YTHDF2-NOTCH1 pathway (28). Endothelial fragmentation reduces nitric oxide bioavailability, disrupts barrier function, and increases susceptibility to ferroptosis; empagliflozin and USP33-dependent stabilization of ATG7 protect the diabetic microvasculature by restraining DRP1/FIS1-mediated remodeling and restoring autophagy (14, 31).

These cell-specific effects can converge clinically as impaired myocardial perfusion, increased myocardial stiffness, and diastolic dysfunction. A treatment that improves cardiomyocyte ATP production but leaves fibroblast or endothelial pathology unchanged may therefore produce incomplete functional recovery. This is a key reason to include perfusion, fibrosis, and cell-resolved endpoints in addition to ejection fraction.

## 5 Therapeutic strategies targeting mitochondrial dynamics

### 5.1 Restraining pathological fission

Several interventions reduce excessive fission without directly targeting the DRP1 GTPase. Empagliflozin activates AMPK and suppresses DRP1-mediated microvascular fragmentation (14); melatonin engages SIRT1-PGC-1α signaling (15); fasudil limits Rho-associated protein kinase 1 (ROCK1)-DRP1 signaling (40); and mammalian sterile 20-like kinase 1 (Mst1) deletion attenuates DRP1-associated remodeling (41). More recent genetic studies suggest that Piezo1, RCAN1, DUSP26, FIS1, OTUD1, VCPIP1, and USP33 are upstream nodes that enable cell-selective modulation (16–19, 21, 24, 31).

Therapeutic interventions only need to reverse pathological excessive fission rather than completely abolishing physiological DRP1 activity. Basal DRP1 activity is required for mitochondrial distribution and mitophagy, and its chronic suppression may retain damaged organelles and impair cardiac adaptation. Convincing preclinical studies therefore combine target perturbation with the restoration of respiratory function, mitophagic flux, cell survival, and global cardiac function, rather than focusing solely on morphological improvement.

### 5.2 Reinforcing fusion, cristae integrity, and metabolic signaling

Fusion-supporting approaches act through several convergent pathways. Nicotinamide riboside increases MFN2-dependent fusion through sirtuin 1- peroxisome proliferator-activated receptor gamma coactivator 1α- peroxisome proliferator-activated receptor α (SIRT1-PGC-1α-PPARα) signaling (42). B-type natriuretic peptide and leptin promote OPA1-dependent fusion through protein kinase G (PKG)-STAT3 signaling and coordinated suppression of cGAS-STING activation, respectively (23, 43). Heparan sulfate-binding domain deleted fibroblast growth factor 1 (FGF1*Δ*HBS) restores mitochondrial homeostasis through AMPK- nuclear receptor subfamily 4 group A member 1 (Nur77) signaling (44), while exercise activates the fibroblast growth factor 21-sirtuin 3 (FGF21-SIRT3) axis to preserve mitochondrial integrity and cardiac function (45).

These interventions are advantageous because they couple mitochondrial morphology with substrate metabolism, cristae maintenance, and antioxidant capacity. However, increased fusion is not automatically beneficial: persistent fusion can impede segregation of irreversibly damaged mitochondrial segments. Translation will require pharmacodynamic markers that demonstrate restoration of dynamic flux and respiratory reserve rather than a simple increase in MFN/OPA1 protein abundance.

### 5.3 Natural products and multi-component interventions

Natural products should be evaluated under the same mechanistic framework as conventional pharmacological agents. Punicalagin promotes OPA1-dependent fusion through protein tyrosine phosphatase 1B (PTP1B)-STAT3 signaling, and OPA1 loss attenuates its protective effect (46). Paeonol similarly promotes OPA1-mediated fusion through casein kinase 2α (CK2α)-STAT3 signaling (47). The multi-component formulation Fufang Zhenzhu Tiaozhi improves abnormal lipid metabolism together with mitochondrial dynamics in diabetic mice (48). These studies provide rigorous mechanistic evidence (OPA1 dependency, upstream pathway validation, and functional rescue assays) that are more informative than classifying therapies solely as “herbal”.

Nevertheless, variations in extract composition, bioavailability, batch consistency, drug exposure, and off-target effects require rigorous characterization. Active component identification and orthogonal functional validation are required before claiming direct targeting of mitochondrial dynamics. The same evaluation criteria apply to synthetic compounds such as RTA 408, in which Nrf2 activation and NF-κB inhibition contribute to the restoration of fission-fusion balance (27).

### 5.4 Current research stage and clinical implications

Most mitochondrial dynamics-targeted interventions remain confined to cellular and rodent models. Current human evidence primarily includes observational myocardial analyses, *ex vivo* tissue studies, and emerging human engineered cardiac tissue models (6, 7, 49). Empagliflozin is clinically established for diabetes and heart failure, but its mitochondrial fission-modulating mechanism has not been validated as a prospectively defined pharmacodynamic endpoint in dCM clinical trials. Currently, no pharmacological agent is specifically approved for dCM treatment via direct modulation of DRP1, MFN1/2, or OPA1.

Accordingly, the translational priority is not to directly advance mitochondrial morphological findings into clinical trials. Instead, it lies in identifying dCM endotypes with activated mitochondrial dynamic pathways, validating target engagement in clinically relevant human models, and selecting therapies with favorable systemic metabolic and cell-specific cardiac profiles. Table 1 summarizes the evidence grade and mechanistic characteristics of representative studies. These programs should pair target-engagement markers with cell-resolved endpoints for energetics, redox stress, regulated cell death, mitophagic flux, fibrosis, and microvascular function.

**Table 1:** Representative studies linking regulatory pathways to mitochondrial fission, fusion, and related mitochondrial quality-control processes in diabetic cardiomyopathy across experimental stages.

Study (reference)	Cardiac cell type/sample	Key regulator(s)	Upstream pathway or stimulus	Effect on mitochondrial dynamics/quality control	Downstream pathological effect	Tested intervention or translational implication	Model/research stage
Montaigne et al. (6)	Human right-atrial myocardium	MFN1	Type 2 diabetes; HbA1c-associated metabolic burden	MFN1 downregulation, mitochondrial-network fragmentation, and impaired respiration	Myocardial oxidative stress and intrinsic contractile dysfunction	No intervention tested; provides human evidence linking impaired fusion-associated remodeling to dysfunction	Human observational/*ex vivo*
Zhou et al. (14)	Cardiac microvascular endothelial cells	AMPK-DRP1	Hyperglycemia; empagliflozin increases the AMP/ATP ratio and activates AMPK	DRP1-Ser616 phosphorylation down and DRP1-Ser637 phosphorylation up; excessive fission restrained	mtROS, senescence, barrier injury, and perfusion defects reduced	Empagliflozin	*In vitro* + animal
Hu et al. (12)	Cardiomyocytes	PPARα-MFN2	Diabetic metabolic stress with suppression of the PPARα-MFN2 axis	MFN2 loss promotes fragmentation; MFN2 restoration re-establishes fusion and network integrity	Mitochondrial dysfunction, ventricular remodeling, and impaired cardiac performance	MFN2 restoration or activation of the PPARα-MFN2 axis	*In vitro* + animal
Wu et al. (20)	Cardiomyocytes	Orai1-Ca2+-ERK/calcineurin-DRP1	High-glucose-induced Orai1-dependent Ca2 + entry	DRP1-Ser616 phosphorylation and Ser637 dephosphorylation increase mitochondrial fission	Mitochondrial dysfunction and cardiomyocyte hypertrophy	Orai1 or DRP1 inhibition	*In vitro* + animal
Fu et al. (46)	Cardiomyocytes	PTP1B-STAT3-OPA1	High glucose; punicalagin inhibits PTP1B and activates STAT3	OPA1 transcription and OPA1-dependent mitochondrial fusion increase	Mitochondrial oxidative stress, hypertrophy, fibrosis, and cardiac dysfunction reduced	Punicalagin; OPA1 dependence confirmed by loss-of-function experiments	*In vitro* + animal
Hu et al. (42)	Cardiomyocytes	SIRT1-PGC-1alpha-PPARα-MFN2	NAD + depletion; nicotinamide riboside restores NAD + and activates SIRT1	MFN2-dependent mitochondrial fusion increases	Mitochondrial ROS and apoptosis reduced; energetics and cardiac function improved	Nicotinamide riboside	*In vitro* + animal
Chang et al. (43)	Cardiomyocytes	NPRA-PKG-STAT3-OPA1	Early compensatory BNP signaling in diabetes	STAT3-dependent OPA1 transcription and mitochondrial fusion increase	Mitochondrial oxidative injury and respiratory dysfunction reduced	B-type natriuretic peptide/PKG signaling	*In vitro* + animal
Ma et al. (22)	Cardiomyocytes	mtROS-mtDNA-cGAS-STING	Palmitate-induced lipotoxicity and mitochondrial membrane injury	Cytosolic mtDNA release and mitochondrial-quality-control failure; fission/fusion not directly quantified	Innate immune activation, inflammatory myocardial injury, and cardiac dysfunction	STING knockdown or pharmacological STING inhibition	*In vitro* + animal
Shu et al. (16)	Cardiomyocytes and human myocardium	RCAN1-DRP1-Ser616	Long-chain acyl-CoA accumulation and diabetic lipid overload	DRP1-Ser616 phosphorylation, mitochondrial fission, and fragmentation increase	Lipid accumulation, mitochondrial dysfunction, and progression of cardiac dysfunction	RCAN1 knockdown	Human observational/*ex vivo* + *in vitro* + animal
Liu et al. (17)	Cardiomyocytes	DUSP26-FAK-ERK-DRP1	Diabetic stress-induced loss of DUSP26 with FAK-ERK activation	Pathological fission increases; DUSP26 restoration normalizes mitochondrial dynamics	Mitochondrial dysfunction, adverse remodeling, and cardiac dysfunction	DUSP26 restoration or FAK-ERK inhibition	*In vitro* + animal
Liu et al. (28)	Cardiac fibroblasts and human dCM tissue	ALKBH5-m6A-YTHDF2-NOTCH1-DRP1	ALKBH5 loss enhances m6A-dependent YTHDF2 phase separation and NOTCH1 mRNA decay	Fibroblast-specific DRP1-associated mitochondrial fission increases	Fibroblast proliferation, extracellular-matrix deposition, fibrosis, and functional impairment	Fibroblast-targeted ALKBH5 restoration, YTHDF2 knockdown, or NOTCH1 rescue	Human observational/*ex vivo* + *in vitro* + animal
Hao et al. (27)	Cardiomyocytes	Nrf2-NF-κB; DRP1, MFN1/2, OPA1	Hyperglycemia; RTA 408 activates Nrf2 and suppresses NF-κB signaling	Fission-fusion balance restored	Oxidative stress, inflammation, apoptosis, and cardiac dysfunction reduced	RTA 408	*In vitro* + animal
Niu et al. (21)	Cardiomyocytes	Piezo1-ERK1/2-DRP1	Diabetes-associated mechanical and Ca2 + stress with Piezo1 activation	ERK/DRP1-dependent fission increases; cardiomyocyte-specific Piezo1 deletion restores dynamics	Mitochondrial injury, fibrosis, and cardiac dysfunction	Cardiomyocyte-specific Piezo1 deletion as genetic proof of concept	*In vitro* + animal
Luo et al. (24)	Cardiomyocytes	FIS1-DRP1-mtROS-NLRP3	Palmitate-induced lipotoxicity	FIS1-DRP1 interaction increases mitochondrial fragmentation	mtROS accumulation and NLRP3-dependent pyroptosis	FIS1 knockdown or disruption of the FIS1-DRP1 interaction	*In vitro* + animal
Zhang et al. (23)	Cardiomyocytes	Leptin-OPA1-cGAS/STING	Leptin signaling during diabetic stress	OPA1-mediated fusion increases; mtDNA release and cGAS-STING activation decrease	Inflammatory signaling and adverse cardiac remodeling reduced	Leptin; OPA1/cGAS-STING axis	*In vitro* + animal
Cai et al. (49)	Human pluripotent-stem-cell-derived cardiomyocytes and engineered heart tissue	Lipotoxic stress; empagliflozin-responsive metabolic pathways	Complex diabetic milieu with a dominant high-fat component	Mitochondrial and contractile abnormalities improve; fission/fusion events are not directly quantified	Cardiomyocyte death and contractile dysfunction reduced	Empagliflozin	Engineered human tissue
Han et al. (18)	Cardiomyocytes	OTUD1-AMPKalpha2-CAMKK2	OTUD1-mediated removal of K63-linked ubiquitin from AMPKalpha2 at K60/K379	AMPK activation, oxidative phosphorylation, and mitochondrial function are impaired; fission/fusion not directly established	Cardiomyocyte hypertrophy, fibrosis, oxidative stress, and cardiac dysfunction	Cardiomyocyte-specific OTUD1 deletion; OTUD1 inhibition as a candidate strategy	*In vitro* + animal
Jiang et al. (25)	Cardiomyocytes and human myocardium	GULP1-IKIP-IKKbeta/NF-kappaB-OPA1	GULP1 loss permits IKIP-mediated inhibition of IKKbeta-dependent NF-kappaB signaling	OPA1 expression and fusion are impaired; GULP1 restoration normalizes mitochondrial morphology	Fatty-acid oxidation and ATP production improve; lipid overload, oxidative stress, apoptosis, and fibrosis decrease	GULP1 restoration; context-specific modulation of the IKIP-NF-kappaB-OPA1 axis	Human observational/*ex vivo* + *in vitro* + animal
Han et al. (19)	Cardiomyocytes	VCPIP1-AMPKgamma1-LKB1	VCPIP1-mediated K63-linked deubiquitination of AMPKgamma1 at K234	AMPK subunit assembly, Thr172 phosphorylation, and mitochondrial respiration are impaired; fission/fusion not directly established	Metabolic dysfunction, hypertrophy, fibrosis, and impaired cardiac function	Cardiomyocyte-specific VCPIP1 deletion; VCPIP1 inhibition as a candidate strategy	Secondary human dataset + *in vitro* + animal
Chen et al. (31)	Cardiac microvascular endothelial cells	USP33-ATG7-FIS1	USP33 stabilizes ATG7 by limiting K63-linked ubiquitination	ATG7-mediated autophagic degradation of FIS1 restrains endothelial mitochondrial fission	Endothelial mitochondrial dysfunction and cardiac microvascular injury reduced	USP33 restoration or targeting the ATG7-FIS1 axis	*In vitro* + animal
Lu et al. (7)	Human left-ventricular myocardium	ACSL1/BNIP3L; lipid-metabolism-mitophagy-ECM modules	Integrated transcriptomic, proteomic, and metabolomic remodeling in the diabetic heart	Mitophagy and mitochondrial quality control are impaired; fission/fusion not directly measured	Coupled abnormalities in fuel metabolism, mitochondrial quality control, and extracellular-matrix remodeling	Biomarker and target prioritization; no intervention tested	Human observational/*ex vivo*

AMPK, AMP-activated protein kinase; BNP, B-type natriuretic peptide; dCM, diabetic cardiomyopathy; DRP1, dynamin-related protein 1; ECM, extracellular matrix; FIS1, fission 1; MFN1/2, mitofusin 1/2; mtDNA, mitochondrial DNA; mtROS, mitochondrial reactive oxygen species; OPA1, optic atrophy 1; PPARα, peroxisome proliferator-activated receptor alpha.

## 6 Future directions

### 6.1 Single-cell and spatial profiling of cell-specific pathways

Single-cell RNA sequencing has already identified cardiomyocyte, fibroblast, endothelial, and immune-cell programs associated with diabetic fibrosis, but transcript abundance cannot directly reflect organelle morphology or protein functional activity (8). Future studies should integrate single-nucleus RNA sequencing with spatial transcriptomics, phosphoproteomics, ubiquitinomics, and cell-type-resolved metabolomics. A priority is to determine whether specific cardiomyocyte subtypes, such as oxidative, glycolytic, stressed, or conduction-related populations, exhibit distinct DRP1/MFN/OPA1 expression patterns and differential therapeutic susceptibility.

Lineage-specific knockout or knock-in models should be complemented with human validation. For example, the fibroblast ALKBH5-NOTCH1 pathway and endothelial USP33-ATG7-FIS1 pathway should be evaluated independently of cardiomyocyte signaling. Spatial analysis is particularly important because subendocardial, perivascular, and fibrotic regions may exhibit distinct substrate availability, oxygen tension, and mechanical stress profiles.

### 6.2 *In vivo* imaging and quantitative assessment of mitochondrial dynamics

Most current studies infer mitochondrial dynamics based on fixed microscopic or electron micrographic imaging. *In vivo* two-photon imaging, intravital microscopy, and photoconvertible mitochondrial reporters enable longitudinal quantification of mitochondrial fission, fusion, transport, and turnover within the intact heart. Combining these tools with cardiac-gated imaging and fluorescent reporters for membrane potential, ROS, calcium homeostasis, and mitophagy can distinguish genuine dynamic restoration from static mitochondrial enlargement or delayed clearance.

Three-dimensional electron microscopy remains a powerful tool for resolving cristae structure and mitochondrial network architecture at nanometer resolution (13). Correlative light-electron microscopy coupled with machine-learning segmentation bridges dynamic mitochondrial flux and ultrastructural morphology. Standardized morphological metrics, including mitochondrial branch length, network connectivity, sphericity, and cristae density, alongside dynamic event rates, improve cross-laboratory comparability.

### 6.3 Cell-selective and mitochondria-targeted delivery

Systemic modulation of DRP1, AMPK, or autophagy may exert off-target effects on skeletal muscle, liver, kidney, immune cells, and systemic vasculature. Accordingly, translational strategies can leverage cardiomyocyte- or endothelial-selective lipid nanoparticles, peptide-decorated nanocarriers, cardiac promoter-driven adeno-associated viral vectors, and mitochondria-penetrating peptides. Nucleic acid-based strategies, including siRNA, antisense oligonucleotides, and mRNA, are well-suited for targeting upstream regulators (e.g., RCAN1, OTUD1, VCPIP1, USP33) upon achieving optimal dose titration and tissue specificity.

Novel delivery systems require rigorous evaluation of biodistribution, mitochondrial uptake, immunogenicity, proarrhythmic risk, and chronic toxic profiles. Given the indispensable role of physiological mitochondrial fission, reversible or feedback-responsive delivery strategies are preferable to constitutive genetic suppression.

### 6.4 Biomarkers and clinical trial design

A clinically valuable biomarker ought to detect pathway activation prior to irreversible fibrosis and shift following target engagement. Candidate biomarker panels consist of circulating cell-free mitochondrial DNA, mitochondrial-derived peptides, cardiomyocyte- or endothelial-originated extracellular vesicles carrying mitochondrial dynamics proteins, acylcarnitine and lipid metabolic signatures, as well as imaging readouts reflecting myocardial energetics and perfusion. Single biomarkers rarely yield sufficient specificity; multimodal biomarker panels need validation in rigorously phenotyped dCM cohorts and patients with adjudicated competing cardiac disorders.

Early-phase clinical trials may enroll enriched patient subgroups characterized by diabetes, diastolic dysfunction, impaired myocardial energetics and consistent circulating or imaging biomarker signatures. Prespecified endpoints should include target engagement, exercise capacity, diastolic function, myocardial perfusion, fibrosis, and safety, not ejection fraction alone. Sex, age, diabetes duration, renal function, obesity, and concomitant SGLT2 inhibitor or GLP-1 receptor agonist therapy should be treated as biological variables rather than nuisance covariates.

### 6.5 Methodological standards and unresolved questions

The field requires consensus regarding model selection, metabolic phenotyping, and mitochondrial dynamics assessment. Studies should report metabolic profiles, diabetes duration, sex, age, cardiac loading status, and cellular sources of analysis (cardiomyocytes, fibroblasts, endothelial cells, or whole heart). Morphological assessment should be integrated with assays of respiration, ATP production, membrane potential, calcium handling, ROS turnover, mitophagic flux, and cardiac function. Fibrosis and myocardial perfusion should be included as parallel translational endpoints.

Several key questions remain unresolved. It remains unclear whether optimal intervention windows precede measurable diastolic dysfunction, whether mitochondrial dynamics endotypes persist throughout disease progression, and whether therapeutic strategies require dual modulation of fission and fusion. Resolving these questions necessitates longitudinal human studies and bidirectional validation across human tissues, engineered heart models, and cell-specific animal systems. Priority should be given to studies that jointly resolve pathway causality, the responsible cardiac cell type, the therapeutic window, and whether target engagement predicts functional benefit.

## 7 Conclusions

Mitochondrial fission–fusion remodeling acts as a mechanistic hub linking diabetic substrate stress to energetic failure, inflammatory signaling, cell death, fibrosis and microvascular dysfunction. Recent dCM-specific studies have moved the field beyond generic excessive fission models, identifying cell-selective pathways controlling calcium entry, mechanosensing, epitranscriptomic regulation, ubiquitin editing, innate immunity and autophagy. The therapeutic target is not uniform mitochondrial elongation, but restored adaptive dynamic flux, cristae integrity and quality-control throughput. Human multi-omics, cell-resolved perturbation, live imaging, targeted delivery and early biomarkers will determine whether these mechanistic advances translate into precise dCM treatment.

## References

[1] Seferović PM Paulus WJ Rosano G Polovina M Petrie MC Jhund PS. Diabetic myocardial disorder. A clinical consensus statement of the Heart Failure Association of the ESC and the ESC Working Group on Myocardial & Pericardial Diseases. Eur J Heart Fail. (2024) 26(9):1893–903. 10.1002/ejhf.3347 38896048

[2] Jia G Hill MA Sowers JR. Diabetic cardiomyopathy. Circ Res. (2018) 122(4):624–38. 10.1161/CIRCRESAHA.117.311586 29449364 PMC5819359

[3] Ritchie RH Abel ED. Basic mechanisms of diabetic heart disease. Circ Res. (2020) 126(11):1501–25. 10.1161/CIRCRESAHA.120.315913 32437308 PMC7251974

[4] Tan Y Zhang Z Zheng C Wintergerst KA Keller BB Cai L. Mechanisms of diabetic cardiomyopathy and potential therapeutic strategies: preclinical and clinical evidence. Nat Rev Cardiol. (2020) 17(9):585–607. 10.1038/s41569-020-0339-2 32080423 PMC7849055

[5] Heather LC Hafstad AD Halade GV Harmancey R Mellor KM Mishra PK. Guidelines on models of diabetic heart disease. Am J Physiol Heart Circ Physiol. (2022) 323(1):H176–200. 10.1152/ajpheart.00058.2022 35657616 PMC9273269

[6] Montaigne D Marechal X Coisne A Debry N Modine T Fayad G. Myocardial contractile dysfunction is associated with impaired mitochondrial function and dynamics in type 2 diabetic but not in obese patients. Circulation. (2014) 130(7):554–64. 10.1161/CIRCULATIONAHA.113.008476 24928681

[7] Lu Q Tang S Fang S Wu Y Chen L Liang M. Multi-omics profiling of the diabetic human heart reveals coupled dysregulation in lipid metabolism, mitophagy, and extracellular matrix remodeling. Genome Med. (2026) 18(1):102. 10.1186/s13073-026-01668-0 42152039 PMC13353004

[8] Li W Lou X Zha Y Qin Y Zha J Hong L. Single-cell RNA-Seq of heart reveals intercellular communication drivers of myocardial fibrosis in diabetic cardiomyopathy. eLife. (2023) 12:e80479. 10.7554/eLife.80479.sa2 37010266 PMC10110238

[9] Quiles JM Gustafsson ÅB. The role of mitochondrial fission in cardiovascular health and disease. Nat Rev Cardiol. (2022) 19(11):723–36. 10.1038/s41569-022-00703-y 35523864 PMC10584015

[10] Cai C Wu F He J Zhang Y Shi N Peng X. Mitochondrial quality control in diabetic cardiomyopathy: from molecular mechanisms to therapeutic strategies. Int J Biol Sci. (2022) 18(14):5276–90. 10.7150/ijbs.75402 36147470 PMC9461654

[11] Parra V Verdejo HE Iglewski M Del Campo A Troncoso R Jones D. Insulin stimulates mitochondrial fusion and function in cardiomyocytes via the akt-mTOR-NFkappaB-opa-1 signaling pathway. Diabetes. (2014) 63(1):75–88. 10.2337/db13-0340 24009260 PMC3868041

[12] Hu L Ding M Tang D Gao E Li C Wang K. Targeting mitochondrial dynamics by regulating Mfn2 for therapeutic intervention in diabetic cardiomyopathy. Theranostics. (2019) 9(13):3687–706. 10.7150/thno.33684 31281507 PMC6587356

[13] Wang B Dai L Liang H He J Zhou J Guan Y. Mitochondrial ultrastructural pathology in diabetic cardiomyopathy: integrated analysis via scanning electron microscopy and 3D visualization imaging. Cardiovasc Diabetol. (2025) 24(1):331. 10.1186/s12933-025-02884-5 40804395 PMC12345013

[14] Zhou H Wang S Zhu P Hu S Chen Y Ren J. Empagliflozin rescues diabetic myocardial microvascular injury via AMPK-mediated inhibition of mitochondrial fission. Redox Biol. (2018) 15:335–46. 10.1016/j.redox.2017.12.019 29306791 PMC5756062

[15] Ding M Feng N Tang D Feng J Li Z Jia M. Melatonin prevents Drp1-mediated mitochondrial fission in diabetic hearts through SIRT1-PGC1alpha pathway. J Pineal Res. (2018) 65(2):e12491. 10.1111/jpi.12491 29575122 PMC6099285

[16] Shu S Cui H Liu Z Zhang H Yang Y Chen X. Suppression of RCAN1 alleviated lipid accumulation and mitochondrial fission in diabetic cardiomyopathy. Metab Clin Exp. (2024) 158:155977. 10.1016/j.metabol.2024.155977 39053690

[17] Liu C Xu X Sun G Song C Jiang S Sun P. Targeting DUSP26 to drive cardiac mitochondrial dynamics via FAK-ERK signaling in diabetic cardiomyopathy. Free Radic Biol Med. (2024) 225:856–70. 10.1016/j.freeradbiomed.2024.11.006 39510451

[18] Han X Zheng R Zhang J Liu Y Li Z Liu G. Cardiomyocyte OTUD1 drives diabetic cardiomyopathy via directly deubiquitinating AMPKα2 and inducing mitochondrial dysfunction. Nat Commun. (2025) 16(1):6668. 10.1038/s41467-025-61901-z 40683882 PMC12276362

[19] Han X Huang Z Liu G Liu Y Li W Zheng J. VCPIP1 drives diabetic cardiomyopathy by deubiquitinating AMPK*γ*1 and preventing AMPKα-γ subunit assembly in cardiomyocytes. Signal Transduct Target Ther. (2026) 11(1):185. 10.1038/s41392-026-02701-9 42144399 PMC13181061

[20] Wu Q-R Zheng D-L Liu P-M Yang H Li L-A Kuang S-J. High glucose induces Drp1-mediated mitochondrial fission via the orai1 calcium channel to participate in diabetic cardiomyocyte hypertrophy. Cell Death Dis. (2021) 12(2):216. 10.1038/s41419-021-03502-4 33637715 PMC7910592

[21] Niu W Liu X Deng B Hong T Wang C Yan Y. Piezo1 deletion mitigates diabetic cardiomyopathy by maintaining mitochondrial dynamics via ERK/Drp1 pathway. Cardiovasc Diabetol. (2025) 24(1):127. 10.1186/s12933-025-02625-8 40114130 PMC11927149

[22] Ma XM Geng K Law BY-K Wang P Pu YL Chen Q. Lipotoxicity-induced mtDNA release promotes diabetic cardiomyopathy by activating the cGAS-STING pathway in obesity-related diabetes. Cell Biol Toxicol. (2023) 39(1):277–99. 10.1007/s10565-021-09692-z 35235096 PMC10042943

[23] Zhang X Hao C Li T Gao W Ren Y Wang J. Leptin attenuates diabetic cardiomyopathy-induced cardiac remodeling via regulating cGAS/STING signaling and OPA1-mediated mitochondrial fusion. Cell Signal. (2025) 132:111805. 10.1016/j.cellsig.2025.111805 40246132

[24] Luo L Wu Q Xiao Q Chen Y Deng Z Cen C. Lipotoxicity-induced upregulation of FIS1 exacerbates mitochondrial fragmentation and promotes NLRP3-dependent pyroptosis in diabetic cardiomyopathy. Free Radic Biol Med. (2025) 228:183–96. 10.1016/j.freeradbiomed.2024.12.049 39734056

[25] Jiang M-Y Zhang X-H Zhang H-L Hou Y Qi M-Y Zuo Y-X. GULP1 protects against diabetic cardiomyopathy through IKIP/NF-κB-dependent improvement of mitochondrial function. Cardiovasc Diabetol. (2026) 25(1):170. 10.1186/s12933-026-03184-2 42015218 PMC13235008

[26] Wu S Zhou Y Liang J Ying P Situ Q Tan X. Upregulation of NF-κB by USP24 aggravates ferroptosis in diabetic cardiomyopathy. Free Radic Biol Med. (2024) 210:352–66. 10.1016/j.freeradbiomed.2023.11.032 38056575

[27] Hao J Zhou J Hu S Zhang P Wu H Yang J. RTA 408 ameliorates diabetic cardiomyopathy by activating Nrf2 to regulate mitochondrial fission and fusion and inhibiting NF-κB-mediated inflammation. Am J Physiol Cell Physiol. (2024) 326(2):C331–47. 10.1152/ajpcell.00467.2023 38047307

[28] Liu Z-Y Lin L-C Liu Z-Y Song K Tu B Sun H. N6-Methyladenosine-mediated phase separation suppresses NOTCH1 expression and promotes mitochondrial fission in diabetic cardiac fibrosis. Cardiovasc Diabetol. (2024) 23(1):347. 10.1186/s12933-024-02444-3 39342271 PMC11439301

[29] Chen Y Huang J Zhou H Lin J Tao J. Pgam5 aggravates hyperglycemia-induced myocardial dysfunction through disrupting Phb2-dependent mitochondrial dynamics. Int J Med Sci. (2024) 21(7):1194–203. 10.7150/ijms.92872 38818468 PMC11134593

[30] Zhou L Su W Wang Y Zhang Y Xia Z Lei S. FOXO1 reduces STAT3 activation and causes impaired mitochondrial quality control in diabetic cardiomyopathy, diabetes. Obe Metab. (2024) 26(2):732–44. 10.1111/dom.15369 37961034

[31] Chen Y Sun X Li X Guan B Yan X Huang C. USP33 alleviates FIS1-dependent mitochondrial fission and cardiac microvascular injury in diabetic cardiomyopathy via deubiquitinating and stabilizing ATG7. Cell Death Dis. (2026) 17:674. 10.1038/s41419-026-08930-8 42218158 PMC13434780

[32] Wu Y Zhang J Wang W Wu D Kang Y Fu L. MARK4 aggravates cardiac dysfunction in mice with STZ-induced diabetic cardiomyopathy by regulating ACSL4-mediated myocardial lipid metabolism. Sci Rep. (2024) 14(1):12978. 10.1038/s41598-024-64006-7 38839927 PMC11153581

[33] Chen Z Li S Liu M Yin M Chen J Li Y. Nicorandil alleviates cardiac microvascular ferroptosis in diabetic cardiomyopathy: role of the mitochondria-localized AMPK-parkin-ACSL4 signaling pathway. Pharmacol Res. (2024) 200:107057. 10.1016/j.phrs.2024.107057 38218357

[34] Zhao M Shen Z Zheng Z Xu Y Zhang J Liu J. Cardiomyocyte LGR6 alleviates ferroptosis in diabetic cardiomyopathy via regulating mitochondrial biogenesis. Metab Clin Exp. (2024) 159:155979. 10.1016/j.metabol.2024.155979 39038735

[35] Ma S Qin J Zhang Y Luan J Sun N Hou G. Disrupting mitochondrial dynamics attenuates ferroptosis and chemotoxicity via upregulating NRF2-mediated FSP1 expression. Cell Rep. (2025) 44(9):116234. 10.1016/j.celrep.2025.116234 40906557

[36] Wang X- Hu F- Huang H Zou C- Yuan Y Qiao Y- Carnosic acid protects against mitochondrial dysfunction and ferroptosis in myocardial ischemia/reperfusion injury through mediating Mfn2. Eur J Pharmacol. (2026) 1019:178690. 10.1016/j.ejphar.2026.178690 41765272

[37] Ma T Huang X Zheng H Huang G Li W Liu X. SFRP2 improves mitochondrial dynamics and mitochondrial biogenesis, oxidative stress, and apoptosis in diabetic cardiomyopathy. Oxid Med Cell Longevity. (2021) 2021(1):9265016. 10.1155/2021/9265016 PMC8592716 34790288

[38] Zheng H Li W Huang G Zhu H Wen W Liu X. Secreted frizzled-related protein 2 ameliorates diabetic cardiomyopathy by activating mitophagy. Biochim Biophys Acta Mol Basis Dis. (2024) 1870(2):166989. 10.1016/j.bbadis.2023.166989 38101654

[39] Zhang Y Zou R Abudureyimu M Liu Q Ma J Xu H. Mitochondrial aldehyde dehydrogenase rescues against diabetic cardiomyopathy through GSK3β-mediated preservation of mitochondrial integrity and parkin-mediated mitophagy. J Mol Cell Biol. (2023) 15(9):mjad056. 10.1093/jmcb/mjad056 PMC11193060 37771085

[40] Fan X Li X Liu H Xu F Ji X Chen Y. A ROCK1 inhibitior fasudil alleviates cardiomyocyte apoptosis in diabetic cardiomyopathy by inhibiting mitochondrial fission in a type 2 diabetes mouse model. Front Pharmacol. (2022) 13:892643. 10.3389/fphar.2022.892643 35865967 PMC9294374

[41] Feng X Wang S Yang X Lin J Man W Dong Y. Mst1 knockout alleviates mitochondrial fission and mitigates left ventricular remodeling in the development of diabetic cardiomyopathy. Front Cell Dev Biol. (2021) 8:628842. 10.3389/fcell.2020.628842 33553168 PMC7859113

[42] Hu L Guo Y Song L Wen H Sun N Wang Y. Nicotinamide riboside promotes Mfn2-mediated mitochondrial fusion in diabetic hearts through the SIRT1-PGC1alpha-PPARalpha pathway. Free Radic Biol Med. (2022) 183:75–88. 10.1016/j.freeradbiomed.2022.03.012 35318101

[43] Chang P Zhang X Zhang J Wang J Wang X Li M. BNP protects against diabetic cardiomyopathy by promoting OPA1-mediated mitochondrial fusion via activating the PKG-STAT3 pathway. Redox Biol. (2023) 62:102702. 10.1016/j.redox.2023.102702 37116257 PMC10165144

[44] Wang D Yin Y Wang S Zhao T Gong F Zhao Y. FGF1^ΔHBS^ Prevents diabetic cardiomyopathy by maintaining mitochondrial homeostasis and reducing oxidative stress via AMPK/Nur77 suppression. Signal Transduct Target Ther. (2021) 6(1):133. 10.1038/s41392-021-00542-2 33762571 PMC7991671

[45] Jin L Geng L Ying L Shu L Ye K Yang R. FGF21–Sirtuin 3 axis confers the protective effects of exercise against diabetic cardiomyopathy by governing mitochondrial integrity. Circulation. (2022) 146(20):1537–57. 10.1161/CIRCULATIONAHA.122.059631 36134579

[46] Fu F Liu C Shi R Li M Zhang M Du Y. Punicalagin protects against diabetic cardiomyopathy by promoting OPA1-mediated mitochondrial fusion via regulating PTP1B-Stat3 pathway. Antioxid Redox Signal. (2021) 35(8):618–41. 10.1089/ars.2020.8248 33906428

[47] Liu C Han Y Gu X Li M Du Y Feng N. Paeonol promotes OPA1-mediated mitochondrial fusion via activating the CK2alpha-Stat3 pathway in diabetic cardiomyopathy. Redox Biol. (2021) 46:102098. 10.1016/j.redox.2021.102098 34418601 PMC8385203

[48] Yan M Liu S Zeng W Guo Q Mei Y Shao X. The Chinese herbal medicine Fufang Zhenzhu Tiaozhi ameliorates diabetic cardiomyopathy by regulating cardiac abnormal lipid metabolism and mitochondrial dynamics in diabetic mice. Biomed Pharmacother. (2023) 164:114919. 10.1016/j.biopha.2023.114919 37302318

[49] Cai L Zhao Y Li Z Xiao L Wu Y Wang S. A human engineered heart tissue-derived lipotoxic diabetic cardiomyopathy model revealed early benefits of empagliflozin. Adv Sci. (2025) 12(30):e03173. 10.1002/advs.202503173 PMC12376570 40433797

